# Firefighter Health: A Narrative Review of Occupational Threats and Countermeasures

**DOI:** 10.3390/healthcare12040440

**Published:** 2024-02-08

**Authors:** Drew E. Gonzalez, Sarah N. Lanham, Steven E. Martin, Richard E. Cleveland, Thad E. Wilson, Emily L. Langford, Mark G. Abel

**Affiliations:** 1 Department of Kinesiology and Sport Management, Texas A&M University, College Station, TX 77845, USA; dg18@tamu.edu (D.E.G.); semartin@tamu.edu (S.E.M.); 2 First Responder Research Laboratory, Department of Kinesiology and Health Promotion, University of Kentucky, Lexington, KY 40506, USA; sarahlanham@uky.edu (S.N.L.); elangfo1@montevallo.edu (E.L.L.); 3 Department of Leadership, Technology, and Human Development, Georgia Southern University, Statesboro, GA 30458, USA; rcleveland@georgiasouthern.edu; 4 Department of Physiology, College of Medicine, University of Kentucky, Lexington, KY 40536, USA; thad.wilson@uky.edu; 5 Department of Health and Human Sciences, University of Montevallo, Montevallo, AL 35115, USA

**Keywords:** cancer, cardiovascular disease, obesity, physical activity, nutrition, sleep, mindfulness, exposure

## Abstract

Structural firefighters are responsible for protecting properties and saving lives during emergency operations. Despite efforts to prepare firefighters for these hazardous occupational demands, the unfortunate reality is that the incidence of health morbidities is increasing within the fire service. Specifically, cardiovascular disease, cancer, and mental health disorders are among the most documented morbidities in firefighters. Pubmed and Google Scholar search engines were used to identify peer-reviewed English language manuscripts that evaluated firefighters’ occupational health threats, allostatic factors associated with their occurrence, and evidence-based strategies to mitigate their impact. This narrative review provides fire departments, practitioners, and researchers with evidence-based practices to enhance firefighters’ health.

## 1. Introduction

Structural firefighters (referred to as “firefighters” herein) perform dangerous occupational tasks to preserve life and property. Despite the inherent occupational hazards, firefighters are increasingly exposed to agents that affect physical and mental health outcomes. These health issues negatively impact quality of life, contribute to line- and non-line-of-duty deaths, and force retirements. Many of these effects have been previously documented, and our theoretical framework focuses on the role of allostatic loading and how this contributes to deleterious health outcomes among structural firefighters. Other firefighter populations, like wildland firefighters, may have different health concerns, and these issues are beyond the scope of this focused review. Therefore, the aims of this manuscript are as follows: (a) summarize the prevalence and impact of prominent physical and mental health morbidities in the fire service, including cardiovascular disease, cancer, depression, and post-traumatic stress disorder; (b) identify allostatic load factors associated with these health morbidities; (c) and evaluate evidence-based strategies to reduce allostatic load to mitigate or diminish the occupational health threat to enhance firefighters’ health and wellbeing. 

Allostasis has been defined as the body’s ability to maintain physiological stability in the presence of dynamic environmental demands [1]. McEwen and Stellar further describe the allostatic load model as the “wear and tear” the body experiences when repeatedly exposed to stressors [2]. These stressors may be physical or psychological, and the brain’s interpretation of them elicits an allostatic response. Physical stressors may include physical activity, toxic exposures, sleep restriction, reduced energy balance, and poor dietary intake. Psychological stressors include deleterious social interactions and any perceived stress from occupational or life factors. Two of the dominant stress response systems include the hypothalamic–pituitary–adrenal axis (HPA) and sympathetic–adrenal–medullary axis (SAM), which function to release glucocorticoids to provide energy and catecholamines to increase cardiovascular responses, respectively [3]. Chronic exposure to perceived stressors may create deleterious effects on (occupational) performance and injury risk. For instance, research indicates that higher levels of chronic mental stress are associated with reduced skeletal muscle recovery from a resistance training stimulus [4]. Furthermore, this study noted similar trends regarding decreased perceived energy and increased perceived soreness and fatigue [4] among high-stress individuals over 4 days post-resistance training. Regarding allostatic load and occupational performance, Lesniak and colleagues noted an inverse relationship between heart rate variability, an autonomic nervous system index, and timed completion of a simulated fireground test [5]. These findings suggest that firefighters who are in a suboptimal psychophysiological state tend to perform occupational tasks at a slower work rate. Given the magnitude of the allostatic load many firefighters are exposed to, it is critical that a comprehensive set of countermeasures are utilized in an attempt to maintain homeostatic balance, which theoretically yields enhanced health and performance outcomes. These fire service-specific countermeasures include physical activity, dietary intake, sleep hygiene, reduced toxic exposure, and psychological coping strategies and are discussed herein. 

## 2. Materials and Methods

The first objective of this literature search was to identify relevant peer-reviewed articles focused on select firefighter morbidities. The second objective was to identify relevant articles focused on evidence-based countermeasures that could be applied to the fire service. Pubmed, Google Scholar, and fire department websites were used to search for relevant literature. Articles published between 1984 and 2024 were considered for inclusion in this narrative review. Keywords utilized in searches for occupational threats and associated countermeasures included: “firefighter”, “fire service”, “allostasis”, “mental stress”, “resistance training”, “tactical performance”, “cardiovascular disease”, “heart attack”, “sudden cardiac event”, “obesity”, “inflammation”, “oxidative stress”, “physiological stress”, “disease”, “cardiorespiratory fitness”, “exercise clinical testing”, “VO_2max_”, “nutrition”, “dietary intervention”, “dietary supplementation”, “substance use”, “tobacco”, “nicotine”, “alcohol”, “shift work”, “circadian rhythm”, “sleep latency”, “disrupted sleep”, “occupational readiness”, “firefighter”, “emergency response”, “skin cancer”, “cancer prevalence”, “self-contained breathing apparatus”, “personal protective equipment”, “engineering controls”, “heat stress”, “occupational exposures”, “PFAS”, “PAH”, “volitile compounds”, “suicide”, “psychological”, “post-traumatic stress disorder”, “mental health”, and “mindfulness”. Non-English manuscripts were excluded from this review. Abstracts and articles were reviewed for content relevance, currentness, robustness, and overlap. 

## 3. Results

### 3.1. Physical Health Threat: Cardiovascular Disease

Firefighting is well recognized as an unpredictable, hazardous, and demanding occupation. The strenuous physical nature of the occupation, psychological stress, and deleterious lifestyle factors increase firefighters’ risk of cardiovascular disease and premature mortality due to sudden cardiac events (SCEs) [6,7,8]. In fact, organizations such as the Federal Emergency Management Agency (FEMA) and the National Fire Protection Association (NFPA) have indicated that SCEs are responsible for the leading cause of on-duty deaths over the past 40 years (≈45–50%) [9,10,11,12,13,14]. In 2020, the NFPA noted that 29 firefighters died due to on-duty SCEs [14]. Nearly half of the reported deaths were related to overexertion and stress, which plays a critical role in the pathophysiologic mechanisms that manifest into cardiovascular disease. The growing body of literature suggests fire suppression activities markedly exacerbate the risk of SCEs by 10- to 100-fold in comparison to non-emergency-related duties [15]. Importantly, “fire suppression activities’’ encompass commonly performed fireground tasks, such as forcible structure entry, structure ventilation, dry and charged hose deployment, and search and rescue operations, with or without added environmental conditions (i.e., heat, smoke, and chemical exposure) [6]. Therefore, a wide range of fireground tasks and stressors can potentially trigger a cardiac event. 

The austere environments place firefighters at an increased risk of acute myocardial infarction [16]. Firefighters who respond to a structure fire emergency often experience a pronounced activation of SAM [7,8,16], leading to increases in heart rate and blood pressure prior to reaching the fireground. Upon arrival, firefighters are faced with extreme heat conditions (50–100 °C), in addition to the physically and psychologically demanding nature of the fireground. Heat stress per se increases heart rate, cardiac contractility, and myocardial oxygen demand [17]. Heat stress combined with exercise and the load carriage requirements of firefighting (i.e., encapsulating personal protective equipment and self-containing breathing apparatus; Mass: ≈26 kg) can produce increased arterial stiffness and hemodynamic responses [18] and ultimately increase the risk of SCEs. Several authors have [16,19] identified pathogenic SCE mechanisms that may occur during fire suppression activities. Heat stress per se decreases central blood volume, left-ventricular end-diastolic volume, and pulmonary capillary wedge pressure [20]. Combined with the physical exertion of firefighting, there is decreased plasma volume due to perspiration [21]; decreased stroke volume [19]; increased blood viscosity [22]; activation of platelets; increased thrombus formation; impaired vascular function; and ultimately, the promotion of myocardial ischemia and injury [16].

**Figure 1:**
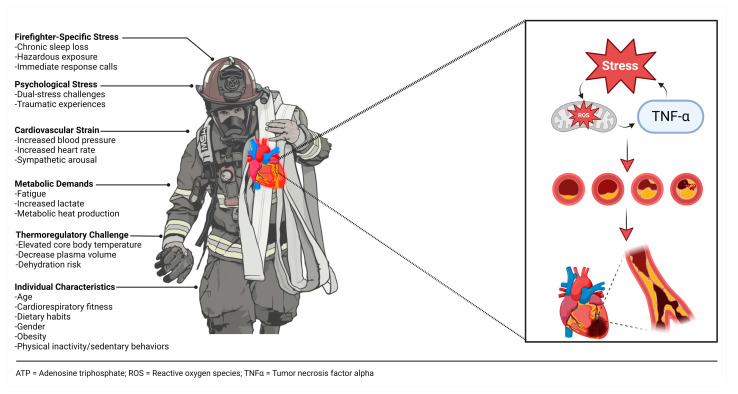
Factors associated with increased cardiovascular strain and subsequent risk of sudden cardiac event among firefighters.

Indeed, factors associated with cardiovascular disease risk and suffering from an on-duty SCE are multifaceted (Figure 1). Acute and chronic exposure to oxidative stress and inflammation may play a key mechanistic role in cardiovascular disease risk [23,24]. Oxidative stress, the imbalance of reactive oxygen species and antioxidants, has been implicated in the development of cardiovascular disease. Firefighting increases the metabolic stress placed on the firefighter, which leads to an increase in free radical production. Additionally, inflammation may play a critical role in vascular dysfunction [25]. Pro-inflammatory cytokines, such as tumor necrosis factor-α and interleukin-6, are elevated following engagement in fire suppression activities [23,26,27,28,29]. Moreover, Huang et al. noted that these pro-inflammatory cytokines are elevated in response to mental and physical stress conditions [30]. Although acute exposure to oxidative stress and inflammation may be advantageous for adaptation, chronic exposure leads to insulin resistance, impaired glucose tolerance, and cardiovascular dysfunction [30].

Obesity is a risk factor for cardiovascular disease as it elevates the likelihood of succumbing to a significant cardiac event by 1.5 to 6.6 times [31]. Indeed, about 75% of firefighters are classified as overweight or obese (≈75%) [32,33,34]. Obesity is associated with lower levels of cardiorespiratory fitness, as well as clusters of other cardiovascular disease risk factors [32,33,34,35,36]. Evidence indicates that firefighters engage in sedentary behaviors and poor dietary practices, which further exacerbates the risk of premature mortality due to cardiovascular disease [37]. In addition, the prevalence of obesity and overweight among firefighters increases the risk of musculoskeletal injuries and reduces the ability to optimally perform critical occupational tasks [32,38,39]. Unfortunately, firefighters are subject to weight gain over the course of their careers. Recent reports suggest that young firefighters are starting their careers with higher body mass index values and arterial blood pressures, poor metabolic profiles, and reduced fitness status [8,40,41]. Ultimately, the prevalence of obesity and overweight, reduced fitness levels, and increased cardiovascular strain create a dire issue for the safety of firefighters.

### 3.2. Physical Health Countermeasure: Clinical and Fitness Assessments

Given the substantial cardiovascular disease issue in the fire service, it is important to utilize clinical assessments to evaluate cardiovascular disease risk. It is well established that higher cardiorespiratory fitness (CRF) levels are inversely associated with cardiovascular disease risk and all-cause mortality [42,43,44]. Maximal oxygen consumption (VO_2max_) tests using a graded exercise test on a treadmill or cycle ergometer with 12-lead electrocardiogram (ECG) monitoring can be used to assess firefighters’ CRF, as well as the risk of SCEs. Importantly, clinical exercise testing (i.e., cardiorespiratory maximal exercise assessments coupled with an ECG) can be used to detect latent cardiovascular disease among asymptomatic individuals or provide prognostic indices [45]. In addition, clinical exercise testing can be used to assess exercise tolerance and discern patterns of disease progression [46]. These clinical assessments are recommended as essential procedures to be included in fire department annual medical exams [47]. As such, greater CRF is associated with reduced blood pressure [48], reduced arterial stiffness [49], and less desirable levels of total cholesterol, triglycerides, low-density lipoprotein cholesterol, insulin resistance, C-reactive protein, and advanced oxidation protein products [35,36,50,51,52]. In addition, higher CRF is associated with lower body mass index (BMI), waist circumference, and percent body fat [53,54,55]. Maintaining higher levels of CRF may also be an important factor in attenuating stress levels among tactical operators [30]. 

The NFPA and researchers have recommended that firefighters should maintain a minimum CRF standard of approximately 42 mL/kg/min in order to meet occupational demands [37,47]. One commonly used protocol to estimate CRF levels among firefighters is the Wellness–Fitness Initiative (WFI) treadmill protocol, which is based upon the following equation: VO_2max_ = 56.981 + (1.242 × TT) − (0.805 × BMI), whereas total time (TT) is the time required to reach 85% maximal heart rate (208 − (0.7 × age) × 0.85) [56]. Interestingly, Dolezal et al. concluded that the WFI protocol had a tendency to overestimate CRF among those who were considered less fit while underestimating those who were considered high fit (i.e., 11% error margin) [56]. Moreover, it was noted that the present policies for measuring CRF among firefighters should be reevaluated, and a more robust approach is likely needed [54,57]. Fire departments are beginning to utilize alternative field-based protocols to assess CRF among firefighters, including maximal effort intermittent shuttle run tests (e.g., 30–15 intermittent fitness test (IFT)), which may simulate the discontinuous physical efforts frequently utilized on the fireground, or continuous shuttle run (beep) tests; both of which allow for multiple firefighters to be tested concurrently. Despite the convenience of these assessments, they rely on estimating firefighters’ CRF. The most accurate way to assess CRF is to measure oxygen consumption with a gas analyzer during a maximal effort on a treadmill or cycle ergometer. CRF assessments might not always be feasible due to expense, time, and requirements for proper exercise testing equipment [56,57,58]. Interestingly, Yang et al. conducted a retrospective longitudinal cohort study among 1104 firefighters from 10 fire departments over a 10-year period [59]. Push-up capacity was found to be inversely associated with 10-year cardiovascular disease risk and significant cardiac events. Specifically, the ability to perform more than 40 push-ups was related to a significant reduction in the risk of experiencing a cardiovascular event compared to individuals who could only complete 10 push-ups. Although muscular endurance may not directly affect the risk of SCEs, push-up muscular endurance capacity appears to be a cost-effective clinical assessment tool; however, more work is needed to further elucidate the relationships of various biomotor abilities with the risk of SCEs.

### 3.3. Physical Health Countermeasure: Exercise

The recommended amount of physical activity to derive health benefits for adults is to accumulate 150 min per week of moderate-to-vigorous physical activity and perform two strength training sessions per week [60]. Unfortunately, only about 20% of firefighters achieve this recommendation [61]. Furthermore, the NFPA recommends that one hour of on-duty time is provided for fitness and wellness activities [62]. Unfortunately, only 27% of United States fire departments provide on-duty exercise time and conduct annual health screenings [63]. In addition, only about 13% of firefighters from one study reported a compliance rate of 75% of on-duty exercise sessions [64]. The lack of exercise participation has deleterious consequences for firefighter safety and health. Specifically, the NFPA recommends that firefighters maintain an aerobic fitness level of 42 mL/kg/min (i.e., 12 metabolic equivalents) for occupational readiness [65]. However, research from one study of 968 firefighters indicated that 56% of firefighters did not achieve this recommendation [50]. This is concerning given that greater aerobic fitness is associated with the efficient completion of occupational tasks [66] and is associated with favorable cardiovascular health outcomes, including diastolic blood pressure, body fat, triglycerides, and low-density lipoprotein profiles [50]. 

The intensity and frequency for which work or exercise are performed are likely contributing factors to SCEs in the fire service. Specifically, research among other populations indicates that there is a dose-dependent relationship between the risk of sudden cardiac death during or following vigorous exercise and the frequency with which vigorous exercise is habitually performed [67]. Unfortunately, it is not feasible to reduce the intensity of occupational tasks; however, firefighters who perform regular vigorous-intensity exercise may be at a lesser risk of occupational SCEs. Indeed, increased exercise frequency among firefighters is associated with reduced cardiovascular disease risk factors, including lower total cholesterol to high-density lipoprotein cholesterol ratio, triglycerides, and glucose levels [61]. Interestingly, the potential cardioprotective effect of exercise on occupational SCEs may be due to the specificity of cardiovascular strain induced by exercise. That is, research indicates that acute changes in hemodynamic and arterial stiffness responses are similar following firefighting tasks and maximal aerobic exercise [18], further supporting the importance of participation in regular endurance exercise.

Despite the known benefits of strength and conditioning programs to fitness and occupational readiness, these resistance-based programs have also demonstrated efficacy for cardiovascular disease risk factors among firefighters [68,69,70]. For instance, a prospective cohort study was conducted among firefighters, which employed a 4-week training intervention (3 session/wk) consisting of firefighter-specific circuit training or traditional linear/nonlinear periodized resistance training groups [68]. Interestingly, circuit training resulted in improvements in vascular health parameters, such as improved blood pressure and flow-mediated dilation profiles, whereas traditional resistance training did not [68]. Furthermore, Pawlak and coworkers demonstrated that performing 12 weeks of on-duty circuit training (2 session/wk) utilizing firefighter equipment as an overload stimulus significantly improved body composition and occupational performance compared to a control group [71]. Thus, exercise interventions utilizing training parameters that stimulate cardiorespiratory and musculoskeletal systems (e.g., circuit training) appear to be effective in improving health and occupational readiness outcomes among firefighters.

### 3.4. Physical Health Countermeasure—Dietary Intake

The International Society of Sports Nutrition (ISSN) recently published a position statement on tactical athlete nutrition [37]. Although little is known regarding specific nutrient intakes and needs, the general recommendations for the provisions and timing of macro- and micronutrients for firefighters are likely similar to those of the general public and/or recreational athletes. In addition, recent reviews of the literature regarding the relationships between diet and cardiovascular disease risk concluded that the existing evidence suggests firefighters are not meeting dietary guidelines [72,73]. A recent cross-sectional study examined the dietary habits of 122 male volunteer firefighters and found the cohort consumed lower daily amounts of fruits and vegetables (2.4 vs. 4.5–5 cups), whole grains (0.8 vs. 6–7 oz), and dietary fiber (16.9 vs. 38 g), and well as higher added sugar intakes, compared to the US recommended daily intakes [74]. Johnson and Mayer also noted firefighters were consuming inadequate amounts of total calories; fiber; vitamins D, E, and K; linolenic and alpha-linolenic fatty acids; potassium; zinc; magnesium; and carbohydrates when compared to the Military Dietary Reference Intakes (MDRI) [75]. It is important to note that MDRIs are specific dietary guidance for the military, and the utilities of MDRIs outside of the military are not well understood. Firefighters likely could benefit from occupation-specific dietary guidance; however, more research is needed to better understand the perceived barriers and dietary habits.

Presently, firefighters are recommended to consider the fueling guidelines outlined in the ISSN position, which stands on tactical athlete nutrition and provides guidance depending on exercise training involvement, as well as consider the Acceptable Macronutrient Distribution Ranges [37]. The foundation of most dietary programs that achieve weight loss is the maintenance of a caloric deficit. Aragon et al. and Donnelly et al. established general recommendations for weight loss, which advise individuals to focus on creating a negative energy balance through exercise expenditure and reduced daily caloric intake [76,77]. In addition, the ISSN recommends that tactical athletes focus on portion control and increase the consumption of nutrient-dense foods and dietary fiber, as well as limit the intake of energy-dense and highly processed foods [37]. To date, there is no one-size-fits-all dietary approach to optimizing firefighter health and fitness. However, there are data to support the potential benefits of the Mediterranean Diet [78,79,80,81] and time-restricted feeding [28,82,83]. Importantly, Yang et al. identified the Mediterranean Diet as a diet of interest to the fire service community due to the potential to yield association with more favorable cardiometabolic profiles compared to the standard American diet [81]. In fact, data from Midwestern firefighters practicing the Mediterranean Diet demonstrate increased high-density lipoprotein cholesterol concentrations, decreased low-density lipoprotein cholesterol concentrations, and less weight gain [81]. Interestingly, Almeida et al. demonstrated that firefighters did not adhere well to the Mediterranean diet (i.e., consumed fewer servings of vegetables as well as greater higher red meat consumption) compared to the civilian control group [84]. 

Time-restricted feeding has garnered increased popularity within the last two decades and can be viewed as strategically manipulating patterns of fasting and feeding [76]. Generally, a 16 h fasting and 8 h feeding pattern is commonly practiced; however, fasting periods can range from 10 to 21 h [85]. To date, only a 14:10 fasting:feeding protocol has been assessed among a cohort of firefighters [82]. Specifically, McAllister et al. found that 6 weeks of a 14:10 fasting:feeding time-restricted feeding protocol resulted in reductions in biomarkers of oxidative stress implicated in the development and progression of cardiovascular disease (i.e., advanced glycation end products and advanced oxidation protein products) [82]. Furthermore, McAllister et al. demonstrated that the same time-restricted feeding protocol for 8 weeks resulted in reductions in the inflammatory (i.e., IL-6 IL-1β) and physiological stress (i.e., cortisol) response to fire suppression activities [28]. Lastly, Gonzalez et al. demonstrated that 7 weeks of a 14:10 fasting:feeding time-restricted feeding protocol does not negatively affect cardiorespiratory or muscle performance parameters (i.e., aerobic capacity, VO_2max_, vertical jump height, one-repetition maximum on bench press and back squat, maximum push-up repetitions) [83]. Based on these findings, it appears that time-restricted feeding is a viable dietary option that can attenuate biomarkers of oxidative stress and inflammation while not hindering physical and occupational performance. There is still a critical need to identify the best dietary approaches in the fire service for the firefighter community to undertake and to mitigate cardiometabolic disease risk. However, it is important to note that due to the unique and extreme occupational demands, there is no one-size-fits-all dietary approach. Based on the existing literature, dietary interventions are needed within the fire service to facilitate improvements in cardiovascular disease risk profiles and enhance occupational readiness.

In terms of dietary supplements that may confer benefits to firefighters, the reader is directed to the recent ISSN position stand regarding tactical athlete nutrition [37]. There are several potentially beneficial dietary supplements that may aid the firefighter, which include creatine, caffeine, beta-alanine, whey protein powders, and essential amino acids, as well as omega-3 fatty acids and L-tyrosine (i.e., for cognitive benefit). However, very little work has been conducted specific to firefighters. To date, two studies have investigated the impacts of caffeine supplementation and firefighter-specific performance and noted increases in core temperature [86] and higher coagulation responses following supplementation [87]. In addition, isolated antioxidants have been purported to confer health benefits to the firefighter. Curcumin, an exogenous antioxidant that may attenuate free radical damage and subsequent oxidative stress, has been studied among a cohort of firefighters conducting live-burn search and rescue drills. In short, McAllister and colleagues assessed the oxidative stress response to live-burn drills among 10 healthy male career firefighters in a randomized, double-blinded, placebo-controlled, crossover design, in which the firefighters performed a routine search and rescue task under different conditions (i.e., no heat, heat with curcumin, heat without curcumin) [88]. Fire-suppressive activities are known to increase free radical damage, which can increase oxidative stress, inflammation, and, ultimately, the risk for cardiovascular disease [23,24,26,28,88,89,90,91]. The curcumin supplementation did not impact the oxidative stress response to the firefighter-specific stressor [88]; however, more research is needed to better understand what supplementation, if any, can aid firefighters in terms of the oxidative stress and inflammatory response to fire-suppressive activities. Consistent with the ISSN position stand, tactical athlete supplementation is an area that requires more attention to identify effective practices for fire personnel that are more efficacious to the fire community.

Hydration strategies have also been highlighted for the tactical athlete [37]. It is important to note that while the athlete recommendation may confer benefits to firefighters, there may be more factors to consider when discussing hydration practice around a live fireground emergency, such as the addition of PPE [92] and added heat [93,94]. Firefighters are advised to replenish fluids at a rate of 0.4–0.8 L/h of activity following exercise or work, as well as include electrolytes when exercising or working in the heat. While more research is warranted among tactical populations, it is generally advisable that firefighters do not rely on thirst as an indicator of hydration status. Rather, they should assess body mass before and after training or events (when possible) and aim to consume 150% of the lost body mass in volume of water [37,95].

### 3.5. Physical Health Countermeasure—Sleep

Circadian rhythm is a complex, natural oscillation of multiple hormone responses and behavior changes across a 24 h period. Circadian alerting signal increases to maintain wakefulness and lessens to promote sleep, whereas lessened circadian altering signal and residual homeostatic sleep drive maintain sleep [96]. Insufficient amounts of acute and chronic sleep is known to have deleterious effects on an individual’s health [97] and wellbeing. Working during traditional sleeping hours may lead to circadian rhythm disruption and/or Shift Work Sleep Disorder (SWSD). SWSD subjects suffer excessive sleepiness or insomnia and an increased risk of gastrointestinal and cardiovascular disease [98]. 

There are two major sleep states: non-rapid eye movement (NREM) and rapid eye movement (REM) sleep. During sleep, the autonomic nervous system displays greater parasympathetic activity than sympathetic activity, except during the REM stage [99]. REM is responsible for processing emotional memory and is only achieved after sufficient slow-wave sleep [99]. An NREM-REM cycle occurs every 90–120 min, with about 3–6 cycles occurring per bout of normal nocturnal sleep. Therefore, the majority of adults require 8 h of sleep per night for optimal function during wakefulness [99,100]. 

Although sleep is highly regulated by circadian rhythm and homeostasis, multiple factors influence the lack of quality sleep hygiene occurring on shift, including emergency response, uncomfortable sleeping quarters, and mental illness [99]. Unfortunately, firefighters have reported disrupted sleep as a result of shift work, with an average of 5 h 21 min in some cases [101]. One night with compromised sleep (i.e., 2 h less than normal) can reduce alertness and occupational performance [100]. Furthermore, an investigation on 24 Australian firefighters highlighted that one night of restricted sleep (i.e., 4 h) may negatively affect reciprocal reaction times [102]. Banks and Dinges report that significant cognitive dysfunction is often present following multiple days of sleep deprivation (i.e., <7 h per night) [103]. Liu and Tanaka noted that individuals with 2 or more instances per week of less than 5 h of sleep per 24 h period were more likely to suffer an acute myocardial infarction than their well-rested peers [104]. Spiegel and colleagues observed compromised endocrine function, reduced carbohydrate metabolism, and increased sympathetic tone among subjects who experienced 6 nights of sleep restriction (i.e., 4 h) [105]. Therefore, restricted sleep may pose an increased risk toward the development of diabetes, hypertension, obesity, and depression [106,107], potentially acting through inflammatory mechanisms and/or overactivation of stress system functions [108,109]. 

Physiologically, the ventrolateral preoptic nucleus (VLPO) and posterior lateral hypothalamus (PLH) control sleep and wakefulness [99]. Caffeine inhibits the activation of VLPO, blocking the ability to promote sleep [110]. Caffeine sensitivity is highly variable between individuals, making precautionary measures widely inconsistent. General guidelines suggest the half-life of caffeine in plasma to be approximately 5 h, with a total plasma clearance rate equating to 0.078 L/h/kg/bw [111]. Peak plasma concentrations are reached 15–120 min following ingestion [112]. Arnaud reported that a dose of 4 mg/kg (280 mg/70 kg body mass) revealed a caffeine half-life of 2.5–4.5 h [113]. Therefore, caffeine consumption is not encouraged before intending to sleep, as consumption can worsen sleep latency, total sleep time, and percentage of time spent in slow-wave sleep (stages 3 and 4) [114].

Alcohol is often used as a relaxant or sleep aid. In non-alcoholic social consumers, alcohol has been shown to decrease sleep latency and increase the quality of NREM sleep during the first half of a sleep session [115]. Unfortunately, using alcohol as a substance to induce sleep is not without fault and disrupts sleep quality during the later stages of sleep, thus disrupting REM and having a negative impact on the homeostatic regulation of sleep [115]. Furthermore, Smith and coworkers found increased sleep disturbances and post-traumatic stress symptom severity to be associated with higher alcohol use among firefighters [116]. 

Nicotine, the primary active ingredient in tobacco, is known to be a highly addictive central nervous system stimulant and depressant. Nicotine centrally activates nicotinic receptors, releasing several neurotransmitters that are involved in sleep regulation (i.e., acetylcholine, dopamine, gamma-aminobutyric acid, norepinephrine, and serotonin), resulting in an arousal-like effect [117,118]. Additionally, as blood-nicotine concentration lessens during sleep, the onset of nicotine withdrawal can disrupt the central nervous system’s ability to keep the individual asleep [118]. Consequently, current smokers suffer from less total sleep time, longer sleep latency, and reduced NREM sleep regulation [117,118,119] compared to non-smokers. Sabanayagam and Shankar suggested that individuals who use both smoke and smokeless tobacco have twice the odds of poor sleep compared to non-tobacco users [119]. The authors further noted that while second-hand smoke exposure may not negatively impact sleep quality among current smokers, it can increase the risk of insufficient sleep among former and non-smokers [119]. The deleterious effects of nicotine use on sleep can be mitigated and often reversed following extended cessation of use [118].

Administering department-wide sleep hygiene education may improve sleep habits among fire personnel [120]. Chung and colleagues elaborated that sleep education generally includes the positive impact of regular exercise, appropriate bedroom arrangement, stress management strategies, regular sleep-wake times, as well as the avoidance of daytime naps and substance use (e.g., caffeine, tobacco, and alcohol) [121]. Substance use is of concern in the fire service as 58% of sampled firefighters attested to binge drinking behavior, 20% confirmed current nicotine use, 14% affirmed hazardous drinking behavior, and 5% stated caffeine overuse [107]. These behaviors negatively impact sleep quality and should be addressed to enhance sleep outcomes. Although some sleep is better than none, multiple sleep cycles during one bout of sleep are superior and cannot be replaced by brief daytime naps [122]. Dissemination of education may encourage discussion for associated policy and culture innovation. A reformation for reducing excessive daytime sleepiness among firefighters may include a frequency reduction of 48 h work shifts [123]. In contrast, direct efforts to lessen sleep disruptions associated with shift work may include eliminating sunlight entry to sleeping quarters, improving air quality, and maintaining a comfortable sleep environment temperature [99,101]. Moreover, Rosekind and colleagues suggest that individuals with difficulty falling asleep do not attempt to force sleep [100]. The authors state that if thirty minutes have passed, abandon the effort and calmly engage in a relaxing activity such as reading [100].

Fire department administrators are encouraged to examine individual perspectives and whole-department culture surrounding sleep hygiene, substance use, and willingness to communicate rest status. Furthermore, departments and practitioners are urged to provide resources and specific support for personnel to assist in efforts to improve sleep hygiene and reduce substance use. 

### 3.6. Physical Health Threat: Cancer

Occupational cancer is now the leading cause of death among firefighters, with firefighters having a 9% greater risk of being diagnosed with cancer and a 14% higher risk of dying from cancer than the general population [124]. Three recent meta-analyses of firefighter cancer studies [125,126,127] indicate consensus in an increased risk of bladder, colorectal, skin (melanoma), lymph node (non-Hodgkin’s lymphoma), prostate, and testicular cancer [128]. The occupational exposure of firefighting (for both career and volunteer and both men and women) was recently classified as “carcinogenic to humans” [125]. 

Firefighters are exposed to aerosolized and topical carcinogenic compounds released during combustion, including per- and polyfluoroalkyl substances (PFAS), polycyclic aromatic hydrocarbons (PAHs), and volatile organic compounds [129,130,131,132,133]. Carcinogenic exposures also include emergency vehicle and equipment diesel exhaust and flame retardants, which are composed of chlorinated tris, polybrominated diphenyl ethers, and PFAS [134]. The most likely routes of entry of these compounds are the respiratory tract and skin. During active fire suppression and knock-down, firefighters wear full PPE, including respirators (self-contained breathing apparatus: SCBA); thus, it is less likely that the respiratory tract is the primary route of entry in this scenario. However, respiratory tract exposure can still occur in the firehouse, from gear, and during overhaul and decontamination procedures. Skin exposure appears to occur in focal areas (neck, jawline, PPE junctions) with full PPE, as PAHs are observed on the skin after fire suppression activities [131]. The skin may even be a route of entry of PFAS directly from fire suppression gear, which can contain high quantities of PFAS in fabrics [135].

The physical exertion and heat stress of firefighting may lead to increased exposure risk for aerosolized and topical carcinogenic compounds. The physical exertion associated with performing firefighting tasks can be significant, especially in full PPE [136]. This exertion leads to large increases in alveolar ventilation [137], which can lead to greater respiratory tract particulate matter and aerosolized compound exposure [138]. Heat stress per se can also increase respiratory rate [139], but the primary organ affected by heat stress responses is the skin. Heat stress increases skin blood flow and vascular volume, which initiates sweating [140,141]. Sweat glands can form a conduit through the skin, allowing contaminants to be more readily absorbed [142,143], as well as increasing the exposed area of skin as the sweat drips and spreads under garments and hydrolyzes chemicals. Direct increases in skin temperature of as little as five degrees increase transdermal absorption by 400% [144], which is quickly observed in a firefighter wearing full PPE [93].

### 3.7. Physical Health Countermeasure: Cancer

Given the prevalence/incidence of occupational cancer among firefighters, it is imperative that countermeasures are utilized in the fire service. To decrease this occupational exposure, several strategies are employed by the fire service: elimination (removing the chemical), substitution (using a less toxic chemical), controls (improving workplace design, training, and educating workers), and PPE (creating a barrier between exposure and person). However, complete mitigation is not possible in the fire service as firefighters are regularly exposed to aerosolized and topical carcinogens during fireground operations. It is also important to note that the “non-occupational” cancer risk may be greater in this population due to the lifestyle factors (e.g., diet, CRF, sleep) described above.

Elimination and substitution are the most effective cancer-reducing strategies, as these involve the removal of potentially toxic exposure [134]. While effective, these changes take time and most often require policy changes and governmental agency involvement. To have this type of large change requires both public and private sector support to be mobilized and then actualized. Control countermeasures fall more directly under the fire service purview, although funding is often needed to implement them. Control measures are typically broken into those involving engineering and design and those involving procedures. Examples of engineering and design controls include fire station vehicle exhaust extraction systems and architectural plans for safety and health in building design [145]. Examples of procedural controls include many fire service policies, such as cleaning and decontamination procedures, as well as crew duty and fire suppression rotations.

PPE should be considered the least effective countermeasure, but it is under the firefighter’s direct control. Firefighter clothing and gear provide the primary protection from thermal and toxic exposures [146]. For aerosolized exposures (toxic or other particulate matter), the use of respiratory protection is paramount. Specifically, SCBAs should be worn throughout all phases of firefighting, not just fire suppression. For topical exposures, gear must be in good working condition and correctly fitted, with specific attention given to junctional areas (e.g., coat to hood, sleeve to glove, pant to boot). Firefighter turnout gear should be decontaminated following each exposure. This may require multiple sets of turnout gear per firefighter in a fire service with a high number of calls and deployments.

### 3.8. Mental Health Threat: Traumatic Events

Despite a decline in national suicide trends, first responders in the United States are more likely to die by suicide than in the line of duty [147]. Concentrating on firefighters alone, a national cross-section of US firefighters found that 47% reported thinking about suicide, 19% admitted planning suicide, and 16% revealed they had attempted suicide during their fire service career [148]. Some analyses estimate that firefighters are 72% more likely to die by suicide than the general U.S. working population [149,150]. Sadly, many factors may impede first responders from receiving help. Such factors include shame and stigma associated with mental health, lack of research and resources, and belief that seeking mental health support will impact employment and/or peer perceptions [147]. 

With such factors serving as obstacles to seeking mental health support, many first responders may employ unhealthy or maladaptive behaviors as coping mechanisms. Mumford, Liu, and Taylor found that in response to continual exposure to stressful situations, first responders were at greater risk of maladaptive behaviors and/or mental health, including smoking, alcohol consumption, post-traumatic stress disorder (PTSD), and even suicidality [151]. As Rudofossi articulated, one of the frequently overlooked aspects of the stressors associated with the work of first responders is also the most insidious [152]. Most individuals recognize, if not understand, the potentially damaging effects of a singular traumatic crisis event. Whether a natural disaster, car accident, terrorist attack, or tragic accident, even the general population acknowledges the subsequent stress following a traumatic incident (i.e., PTSD). Less widely recognized is the cumulative effect of exposure to continual stressful incidents. On the surface, these stressors may seem only slightly disturbing, resulting in them being ignored or dismissed. These stressors are commonly referred to as Potential Traumatic Events (PTEs) and may require a longer time to manifest but carry just as deadly consequences for firefighters.

Even more disheartening is the pervasive, expanding nature of PTEs, as they not only impede the wellness of the firefighter but spill over to affect firefighters’ families as well [153,154]. In their qualitative research exploring the impact of firefighting on the mental health of Canadian firefighters, MacDermid, Lomotan, and Hu outline the unfortunate scope of the consequences of PTEs, stating the following:

“Many firefighters spoke about the impact on their families. […] Reasons given were that they were concerned that their family member might change the way they viewed them as a person because of the kinds of things they were seeing at work. Concurrently, they also wanted their family members to have faith that their role as a firefighter was valuable and that their job was good and important, and they were worried that sharing some of their experiences might undermine their families’ valuation of the job. […] Firefighters acknowledged that the issues that impact their confidence in self and feelings of failure from bad outcomes at work carried over into their feelings within the family. Firefighters also acknowledged awareness and regret that mental health stressors from work carried over into their family life in terms of how they treated family members with less tolerance, irritability, or poor communication. […] They were concerned that taking time off work often compromised their family’s financial stability since sick benefits do not replace usual firefighter income. Many acknowledged the increased risk of divorce in the fire service or had personally experienced divorce and attributed work issues as a contributing factor” (p. 8, 2021) [155].

It would appear that the stressors inherent in firefighting exact a toll that cannot be avoided. Whether a one-time singular crisis event or the culmination of regularly occurring PTEs, the toll must be paid, and the cost appears to be shared between firefighters and their loved ones.

Singular traumatic crises are understood by many as simply “part of the job”. Responses to such crises demand dedicated resources—resources such as personnel, equipment, logistics, financial allocation, and time. Similarly, such crises may demand the attention of the community at large, simultaneously highlighting the firefighters involved, the actions required of them, and the cost(s) associated with such actions. As a whole, a singular crisis event may frequently occur/unfold along a linear, well-publicized “narrative”. Such a narrative has a beginning (i.e., awareness/reporting of the crisis), a present-tense middle (i.e., fire services engaging the crisis), an ending (i.e., resolution of the crisis), and even an epilogue (i.e., community reaction, follow-up, investigation, etc., of the crisis and contributing factors). Non-singular crisis events, or PTEs, regularly encountered during firefighting activities stand in stark contrast as they may not fit into a narrative, may not receive community attention/awareness, and may be more asymmetric than linear in nature. Awareness of these stressors is essential, and the literature has produced various concepts to address their existence and influence. In addition to PTEs [150], other terms addressing the culmination of stressors upon firefighters include “acute stress”, “threatening stress”, and/or “allostatic overload” [156,157].

### 3.9. Mental Health Countermeasure: Mindfulness

Mindfulness is a tool that can be used to attenuate allostatic load. Mindfulness is commonly understood as referring to an individual’s ability to pay attention to and be aware of thoughts, feelings, and sensations occurring in the present moment [158] and has a robust evidence base specifically when administered through Mindfulness-Based Stress Reduction (MBSR) interventions [159]. As a clinically operating construct, mindfulness can be defined as paying attention to the present moment on purpose and without judgment [160]. Mindfulness-Based Interventions (MBIs), whether explicitly MBSR or otherwise, can be stand-alone interventions or easily paired with cognitive theory (CT) or cognitive behavioral theory (CBT) therapies yielding Mindfulness-Based Cognitive Therapy (MBCT) resources and supports [161]. Mindfulness has been evaluated among firefighters, demonstrating beneficial results in combating maladaptive responses to allostatic overloads, such as depression, anxiety, ruminative thinking, and suicide [162,163]. In a noteworthy investigation, Smith et al. explored trait mindfulness among approximately 124 full-time urban firefighters [164]. The study reviewed individual firefighter variables (i.e., age, number of calls responded to, exposure to duty-related stressors, and years of occupational experience) and mental health/resilience support variables (i.e., perceived level of optimism, perceived personal mastery, available social support, and trait mindfulness) to explore the interactions between these variables and maladaptive health symptoms (i.e., PTSD, depression, physical symptoms, and alcohol use). The results indicated that personal mastery and social support were associated with fewer depressive symptoms. More importantly, firefighter mindfulness was negatively correlated to PTSD, depression, negative physical symptoms, and problematic alcohol use, even when controlling for the other mental health/resilience support variables and individual firefighter variables [164]. The research of Smith et al. serves as seminal work regarding the efficacy of MBIs in the fire service [164].

MBIs are flexible in their implementation, providing support across the entire continuum of stressor incidents or PTEs. MBIs can be utilized pre-incident, guiding firefighters with preventative strategies for navigating traumatic events and preventing the development of PTSD. Similarly, MBIs can be deployed during an incident, facilitating present-moment focus and awareness and decreasing the likelihood of disassociation while responding. Finally, MBIs can be offered post-incident, providing firefighters with strategies and exercises focused on managing emotional arousal, addressing negative emotions, and dealing with intrusive and pervasive thoughts or rumination. In this way, successful use of MBIs post-incident can decrease firefighters’ use of unhealthy coping strategies (e.g., unhealthy alcohol consumption).

In addition, MBIs are able to maintain fidelity when delivered through or partnered with other support services. In a study by Smith et al., mindfulness was explored in addition to other supports, yet emerged distinct in its benefits for firefighters [164]. This suggests an ability to align or integrate MBIs with other firefighter supports and resources while requiring minimal investment or structural changes. On a surface level, this may prove extremely beneficial in terms of cost savings (especially for smaller firefighting agencies with restricted budgets) as agencies can incorporate mindfulness practices or interventions within existing training schedules and/or exercises. On a deeper level, this therapeutic flexibility may actively combat the stigma associated with mental health. Even without embedding within other training activities, MBIs may be more readily accepted than other practices traditionally recognized as explicitly associated with mental health (i.e., relaxation techniques) by firefighters [165]. When embedded within regularly established training activities (e.g., incorporating mindful breathing exercises as a part of SCBA breathe-training), MBIs carry the potential to deliver much-needed mental health and resilience support in ways that diffuse social stigma.

### 3.10. Mental Health Countermeasure: Post-Trauma Debriefing

Post-trauma debriefing strategies have been utilized to varying degrees among firefighters for several decades [166] in an effort to reduce the incidence of PTSD and other psychological symptoms. There are several commonly employed debriefing strategies, including critical incident stress debriefing (CISD) and one-to-one debriefing with a peer counselor. CISD utilizes a trained facilitator to discuss feelings and distress experienced by a group of firefighters [167]. Jeannette and Scoboria conducted an investigation to evaluate firefighters’ preference for post-incident interventions while accounting for event severity [167]. Their results indicated that firefighters preferred informally speaking with peers across various types and severity of incidents, whereas firefighters expressed a greater preference for both informal and formal interventions as the incident severity increased [167]. In addition to incident interventions, social support for firefighters is critical in reducing the risk of developing PTSD [168]. A summary of the identified health threats and countermeasures is provided in Table 1.

## 4. Future Directions

It is important to note that this manuscript presented a narrative review of the relevant literature; as such, it provides a gross overview of occupational health threats and applicable countermeasures. Future efforts should employ a systematic review of the literature utilizing defined PRISMA guidelines to enhance the transparency and, thus, quality of the results and conclusions. Additionally, future research in the fire service should continue to identify risk factors associated with deleterious physical and mental health outcomes and evaluate the efficacy of targeted interventions. Increased research emphasis should be placed on understudied populations, including female and volunteer firefighters. 

## 5. Conclusions

The prevalence of firefighters’ physical and mental health morbidities has increased in recent years. The most prevalent physical chronic diseases include cardiovascular, metabolic, obesity, and cancer morbidities, whereas acute and chronic psychological stress and PTSD are the most commonly reported mental health issues. Allostatic load factors that are associated with these morbidities include physical stressors, chronic sleep restriction, poor dietary intake, and social and psychological stressors. A host of programmatic strategies may be implemented to reduce the deleterious effects of these allostatic factors. This work requires a commitment from executive leadership within fire agencies to provide fiscal and time management resources and utilize subject matter experts to implement evidence-based wellness interventions. 

## Figures and Tables

**Table 1:** Description of prominent morbidities within the fire service and potential countermeasures to be utilized by fire departments.

Morbidity	Cardiovascular Disease	Psychological Disorders	Cancer
Countermeasure	Medical and fitness screenings; Regular physical activity; Nutritious dietary intake; Promote sleep hygiene	Practice mindfulness and meditation; Utilize positive coping mechanisms; Immediately address traumatic events	Perform frequent cancer screenings; Maintain PPE; Reduce environmental and behavioral exposures; Eliminate use of carcinogenic foaming retardants

PPE: Personal protective equipment.
